# External Validation of MRI-Based Prostate Cancer Risk Calculators in a Contemporary Pre-Biopsy mpMRI Cohort

**DOI:** 10.3390/jcm15208146

**Published:** 2026-10-20

**Authors:** Hubert Grießner, Sarah K. Luck, Julia K. Peters, Kathrin Olesch, Manuel Schlachter, Lukas Oberhammer, Maximilian Pallauf, Lukas Lusuardi, Philipp N. Haid

**Affiliations:** Department of Urology, Paracelsus Medical University Salzburg, 5020 Salzburg, Austria; h.griessner@salk.at (H.G.); j.peters@salk.at (J.K.P.); k.olesch@salk.at (K.O.); m.schlachter@salk.at (M.S.); l.oberhammer@salk.at (L.O.); m.pallauf@salk.at (M.P.); l.lusuardi@salk.at (L.L.)

**Keywords:** prostate cancer, multiparametric MRI, risk calculator, external validation, clinically significant prostate cancer, decision curve analysis

## Abstract

**Background**: Multiparametric MRI (mpMRI)-integrated risk calculators may improve individualized prediction of clinically significant prostate cancer (csPCa) before biopsy. We externally validated and directly compared the MRI-based Radtke and Alberts risk calculators (Radtke-RC and Alberts-RC) in a contemporary MRI-selected biopsy cohort, assessing discrimination, calibration, and clinical utility. **Methods**: Consecutive patients undergoing pre-biopsy mpMRI followed by combined MRI-targeted and systematic biopsy were retrospectively identified. The primary endpoint was csPCa, defined as ISUP Grade Group ≥ 2. Discrimination was assessed using receiver operating characteristic analysis, calibration using observed-to-expected (O:E) ratios, calibration-in-the-large (slope fixed at 1), calibration slopes, and calibration plots, and clinical utility using decision curve analysis. Radtke-RC validation was restricted to biopsy-naïve patients, while head-to-head comparisons were restricted to biopsy-naïve patients evaluable for both calculators. Threshold probabilities of 12%, 20%, and 30% were examined, with bootstrap confidence intervals for incremental net benefit. **Results**: Of 171 screened patients, seven had no evaluable biopsy outcome. The Radtke-RC validation cohort comprised 144 biopsy-naïve patients, including 55 (38.2%) with csPCa. The Alberts-RC was evaluable in 114 patients; 50 patients could not be assessed because age, prostate volume, or PSA values were outside the online calculator’s permitted input ranges. The common biopsy-naïve cohort comprised 97 patients, including 34 (35.1%) with csPCa. In the model-specific cohorts, AUCs were 0.765 (95% CI 0.682–0.848) for the Radtke-RC and 0.767 (95% CI 0.672–0.861) for the Alberts-RC. In the common cohort, AUCs were 0.774 (95% CI 0.672–0.875) and 0.768 (95% CI 0.661–0.874), respectively, with no statistically significant paired difference (0.006, 95% CI −0.028 to 0.040; *p* = 0.726). Calibration differed substantially: the Radtke-RC overpredicted risk (mean predicted risk 48.2%; O:E 0.727), whereas the Alberts-RC underpredicted risk (18.0%; O:E 1.944). At thresholds of 12%, 20%, and 30%, the Radtke-RC classified zero, seven, and 25 patients below threshold and missed zero, zero, and four csPCa cases, respectively; corresponding values for the Alberts-RC were 42, 64, and 78 patients below threshold, with seven, 12, and 20 csPCa cases missed. Sensitivity analyses indicated less favourable Radtke-RC decision-analytic performance outside the common cohort, whereas restriction to PI-RADS 4–5 patients did not materially alter relative discrimination. **Conclusions**: No statistically significant difference in discrimination between the Radtke-RC and Alberts-RC was detected, although the study was not powered to establish equivalence. Despite similar discrimination, the calculators showed substantially different calibration and threshold-specific clinical consequences. In this MRI-selected population, interpretation of fixed risk thresholds should therefore account for calibration, patient selection, and the trade-off between biopsy avoidance and missed csPCa.

## 1. Introduction

Prostate cancer (PCa) is among the most frequently diagnosed malignancies in men worldwide and remains an important cause of cancer-related morbidity and mortality [1]. Although prostate-specific antigen (PSA) testing facilitates early detection, its limited specificity can lead to unnecessary prostate biopsies and overdiagnosis of clinically insignificant disease [2]. Contemporary diagnostic pathways therefore increasingly aim to identify clinically significant prostate cancer (csPCa) while avoiding invasive procedures in men unlikely to harbour clinically relevant disease.

Multiparametric magnetic resonance imaging (mpMRI) has substantially changed the diagnostic evaluation of men with suspected PCa [3,4]. Standardized assessment using the Prostate Imaging Reporting and Data System (PI-RADS), combined with MRI-targeted biopsy, improves detection of csPCa and may reduce unnecessary biopsies compared with diagnostic strategies based primarily on PSA and systematic biopsy [3,4]. Nevertheless, MRI findings alone do not provide an individualized probability of csPCa, and their predictive value is influenced by patient characteristics, disease prevalence, imaging quality, and reader interpretation [5]. Integrating MRI findings with established clinical predictors may therefore provide more individualized estimates of biopsy-detectable csPCa risk.

Several multivariable risk calculators have been developed for this purpose. Earlier models, including those derived from the Prostate Cancer Prevention Trial and the European Randomized Study of Screening for Prostate Cancer (ERSPC), combined clinical variables such as age, PSA, digital rectal examination, prostate volume, and previous biopsy status [6,7,8,9]. More recently, mpMRI findings have been incorporated into prediction models. Among these are the model developed by Radtke et al. (Radtke-RC) and the MRI-integrated Rotterdam ERSPC model developed by Alberts et al. (Alberts-RC) [10,11]. Both combine PI-RADS assessment with clinical predictors and have subsequently undergone external validation in different populations and diagnostic pathways [12,13,14,15,16,17,18,19].

External validation, however, requires more than assessment of discrimination. Models with similar areas under the receiver operating characteristic curve may generate substantially different absolute risk estimates when transported to populations that differ from their development cohorts. Calibration is therefore particularly important when predicted probabilities are used to guide threshold-based clinical decisions. Decision curve analysis (DCA) provides complementary information by quantifying the clinical consequences of using a prediction model compared with default strategies such as biopsying all or no patients [20]. Recent comparative studies have demonstrated considerable heterogeneity in calibration and clinical utility among MRI-based risk calculators across populations and clinical pathways [15,16,17,18,19]. These observations emphasize that discrimination, calibration, and decision-analytic performance should be interpreted jointly when evaluating prediction models for clinical use [21,22,23].

Pallauf et al. previously externally validated the Radtke-RC and Alberts-RC in a cohort consisting exclusively of men with a previous negative biopsy and demonstrated that their clinical utility was strongly dependent on the selected risk threshold [24]. However, model performance and absolute risk estimates may differ in contemporary populations in which mpMRI has already contributed to the decision to proceed to biopsy. Such MRI-selected cohorts represent a distinct validation setting because patient selection, disease prevalence, PI-RADS distribution, and predictor measurement may differ from those of the original development cohorts and previous validation studies. Moreover, restrictions imposed by an online calculator may determine which patients are evaluable and thereby influence the population available for direct model comparison.

The present study therefore aimed to externally validate the Radtke-RC in biopsy-naïve men and the Alberts-RC in patients eligible for its application, and to directly compare both calculators in a common biopsy-naïve population evaluable for both models. We assessed discrimination, calibration, and clinical utility, with particular emphasis on threshold-specific biopsy avoidance and missed csPCa. In addition, we examined whether selection into the common cohort influenced model performance and performed sensitivity analyses addressing cohort selection and PI-RADS distribution.

## 2. Materials and Methods

This retrospective single-centre external validation study was conducted at Paracelsus Medical University Salzburg, Austria. Consecutive patients undergoing pre-biopsy multiparametric MRI (mpMRI) followed by prostate biopsy between October 2023 and September 2025 were screened. No formal sample-size calculation was performed. Seven of 171 patients had no evaluable biopsy outcome, leaving 164 with an evaluable reference standard. Because the prevalence-adjusted Radtke risk calculator (Radtke-RC) implementation used here was validated in biopsy-naïve men, 20 patients with a previous negative biopsy were excluded from Radtke-RC validation, resulting in 144 biopsy-naïve patients. The Alberts risk calculator (Alberts-RC) was applicable in 114 patients, comprising 97 biopsy-naïve men and 17 men with a previous negative biopsy. In the remaining 50 patients, all required predictors were available, but age (*n* = 43), prostate volume (*n* = 6), or PSA (*n* = 1) was outside the permitted input range of the online calculator. The 97 biopsy-naïve patients evaluable for both models constituted the common cohort.

MRI was assessed according to PI-RADS version 2.1 [4] by radiologists from University Hospital Salzburg and external outpatient practices without centralized re-review. Reader number and experience could not be reconstructed retrospectively. For multiple lesions, the highest PI-RADS category was used. Prostate volume was taken from the routine clinical mpMRI report; the original estimation method was not consistently documented. All patients underwent combined MRI-targeted and systematic fusion-guided biopsy using a transperineal or transrectal approach. Patient-level biopsy route was incompletely documented, precluding route-specific analyses. Where documented, targeted biopsy comprised a median of six cores (IQR 6–6) and systematic biopsy 12 cores (IQR 12–12).

The primary endpoint was clinically significant prostate cancer (csPCa), defined as ISUP Grade Group ≥ 2 on combined biopsy histopathology [25]. No study-specific blinding procedures were applied. Radtke-RC probabilities were calculated using R version 4.4.0 (R Foundation for Statistical Computing, Vienna, Austria) and the published prevalence-adjusted 2019 implementation without modification: logit(p) = −4.5720 + 0.550 × ln(PSA) − 0.020 × prostate volume + 0.034 × age + 0.712 × I(suspicious DRE) + 0.299 × I(PI-RADS 2) + 1.198 × I(PI-RADS 3) + 1.841 × I(PI-RADS 4) + 3.094 × I(PI-RADS 5), with PI-RADS 1 as the reference category [26]. Alberts-RC probabilities were obtained from the Rotterdam ERSPC/SWOP calculator (SWOP—The Prostate Cancer Research Foundation, Reeuwijk, The Netherlands) [11,27]. Risk Calculator 3 was used for biopsy-naïve men and Risk Calculator 4 for men with a previous negative biopsy. Recorded PI-RADS categories, including PI-RADS 1, were entered without recoding. The displayed csPCa probability was recorded as a whole-number percentage and converted to a proportion for analysis. Manual entry and transcription of SWOP outputs were independently verified by two investigators.

Age was summarized as mean ± SD, PSA and prostate volume as median (IQR), and categorical variables as *n* (%). Because the model-specific cohorts overlapped, baseline characteristics were presented descriptively without formal between-cohort hypothesis testing. Discrimination was assessed using empirical unsmoothed ROC curves and AUCs with 95% CIs; paired comparison in the common cohort used DeLong’s method [28]. Calibration was assessed using calibration plots, observed-to-expected ratios, calibration-in-the-large, and calibration slopes. Calibration-in-the-large was estimated by logistic regression with the logit of predicted risk as an offset (slope fixed at 1). Calibration slopes and the plotted logistic calibration curves were estimated from separate models with freely estimated intercepts and slopes. Formal recalibration was not performed because the objective was external validation rather than model updating.

Clinical utility was evaluated by decision curve analysis against biopsy-all and biopsy-none over thresholds of 0–35% [20]. The 12% threshold represented a low decision range in which model-based net benefit approached that of biopsy-all, whereas 20% and 30% were selected descriptively to illustrate increasingly biopsy-averse strategies. Incremental net benefit versus biopsy-all was evaluated using 2000 patient-level bootstrap resamples with percentile 95% CIs. Sensitivity analyses assessed Radtke-RC performance in the full *n* = 144 cohort and Radtke-only *n* = 47 subgroup and repeated paired discrimination analysis in PI-RADS 4–5 patients. Multiple imputation was not performed because Alberts-RC non-evaluability reflected out-of-range inputs rather than missing core predictors. Analyses were performed in R version 4.4.0. Final ethics approval was granted on 27 March 2026 (EK No. 1081/2025); study-specific analyses began only thereafter, and the final REDCap (Research Electronic Data Capture; Vanderbilt University, Nashville, TN, USA) dataset was exported on 21 August 2026. The reporting of this study was retrospectively assessed against the TRIPOD framework for external prediction-model validation [29].

## 3. Results

**Figure 1:**
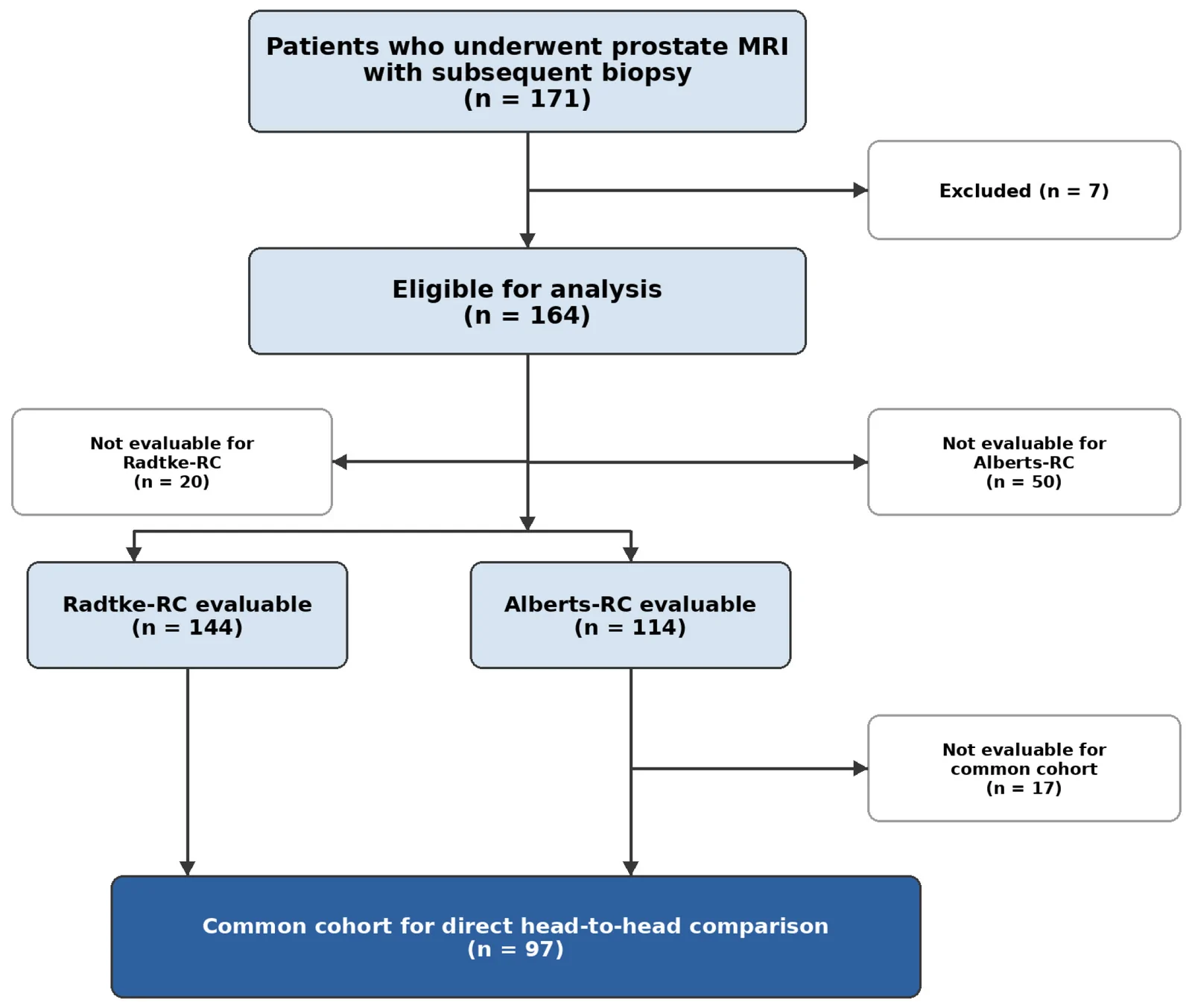
Flowchart of patient selection and formation of the model-specific and common analysis cohorts. Of 171 screened patients, seven were excluded because no evaluable biopsy outcome was available. Among the remaining 164 patients, 20 with a previous negative prostate biopsy were excluded from Radtke-RC validation, leaving 144 biopsy-naïve patients. The Alberts-RC was evaluable in 114 patients, whereas 50 could not be evaluated because age (*n* = 43), prostate volume (*n* = 6), or PSA (*n* = 1) was outside the permitted input range of the online calculator. The Alberts-RC cohort comprised 97 biopsy-naïve patients and 17 patients with a previous negative biopsy. The 97 biopsy-naïve patients evaluable for both calculators constituted the common cohort for direct head-to-head comparison. Abbreviation: RC, risk calculator.

A total of 171 patients were screened for eligibility. Seven had no evaluable biopsy outcome, leaving 164 patients with an evaluable reference-standard outcome. Twenty patients with a previous negative prostate biopsy were excluded from Radtke-RC validation, resulting in a biopsy-naïve Radtke-RC cohort of 144 patients. The Alberts-RC was evaluable in 114 of 164 patients, comprising 97 biopsy-naïve patients and 17 patients with a previous negative biopsy. In the remaining 50 patients, all required Alberts-RC predictors were available, but the online calculator could not be applied because values were outside its permitted input ranges for age (*n* = 43), prostate volume (*n* = 6), or PSA (*n* = 1). The 97 biopsy-naïve patients evaluable for both calculators formed the common cohort for direct head-to-head comparison. Among the remaining 47 biopsy-naïve Radtke-RC patients, Alberts-RC non-evaluability resulted from out-of-range age (*n* = 41), prostate volume (*n* = 5), or PSA (*n* = 1). The patient selection process and cohort composition are illustrated in the flowchart in Figure 1. Baseline characteristics of the Radtke-RC, Alberts-RC, and common cohorts are summarized descriptively in Table 1. Compared with the common cohort, the mutually exclusive Radtke-only subgroup was descriptively older and had higher PSA values and larger prostate volumes, indicating that eligibility for the Alberts-RC selected a clinically distinct subset of biopsy-naïve patients.

Clinically significant prostate cancer was detected in 55 of 144 patients (38.2%) in the Radtke-RC cohort, 41 of 114 (36.0%) in the Alberts-RC cohort, and 34 of 97 (35.1%) in the common cohort. Two patients in the Radtke-RC cohort had PI-RADS 1 findings, including one patient in both the Alberts-RC and common cohorts; neither had csPCa. Both patients with PI-RADS 1 had suspicious DRE findings; however, the complete clinical rationale for proceeding to biopsy could not be retrospectively attributed to a single indication. Recorded PI-RADS categories were retained without analytical recoding, and no patient had a PI-RADS 2 finding. In the Radtke-RC cohort, csPCa was detected in four of 15 patients with PI-RADS 3 (26.7%), 24 of 79 with PI-RADS 4 (30.4%), and 27 of 48 with PI-RADS 5 (56.3%). In the Alberts-RC cohort, corresponding rates were four of 14 (28.6%), 17 of 67 (25.4%), and 20 of 32 (62.5%). In the common cohort, rates were four of 12 (33.3%), 14 of 56 (25.0%), and 16 of 28 (57.1%), respectively. Thus, the csPCa gradient was most pronounced for PI-RADS 5, whereas separation between PI-RADS 3 and 4 was limited.

Among the 55 patients with csPCa in the Radtke-RC cohort, biopsy-specific attribution could be determined in 51. Clinically significant cancer was detected by both MRI-targeted and systematic biopsy in 35 of 51 patients (68.6%), exclusively by MRI-targeted biopsy in nine (17.6%), and exclusively by systematic biopsy in seven (13.7%). Biopsy-specific attribution was unavailable in four patients; these patients remained in the primary analyses because the combined biopsy outcome was known.

**Figure 2:**
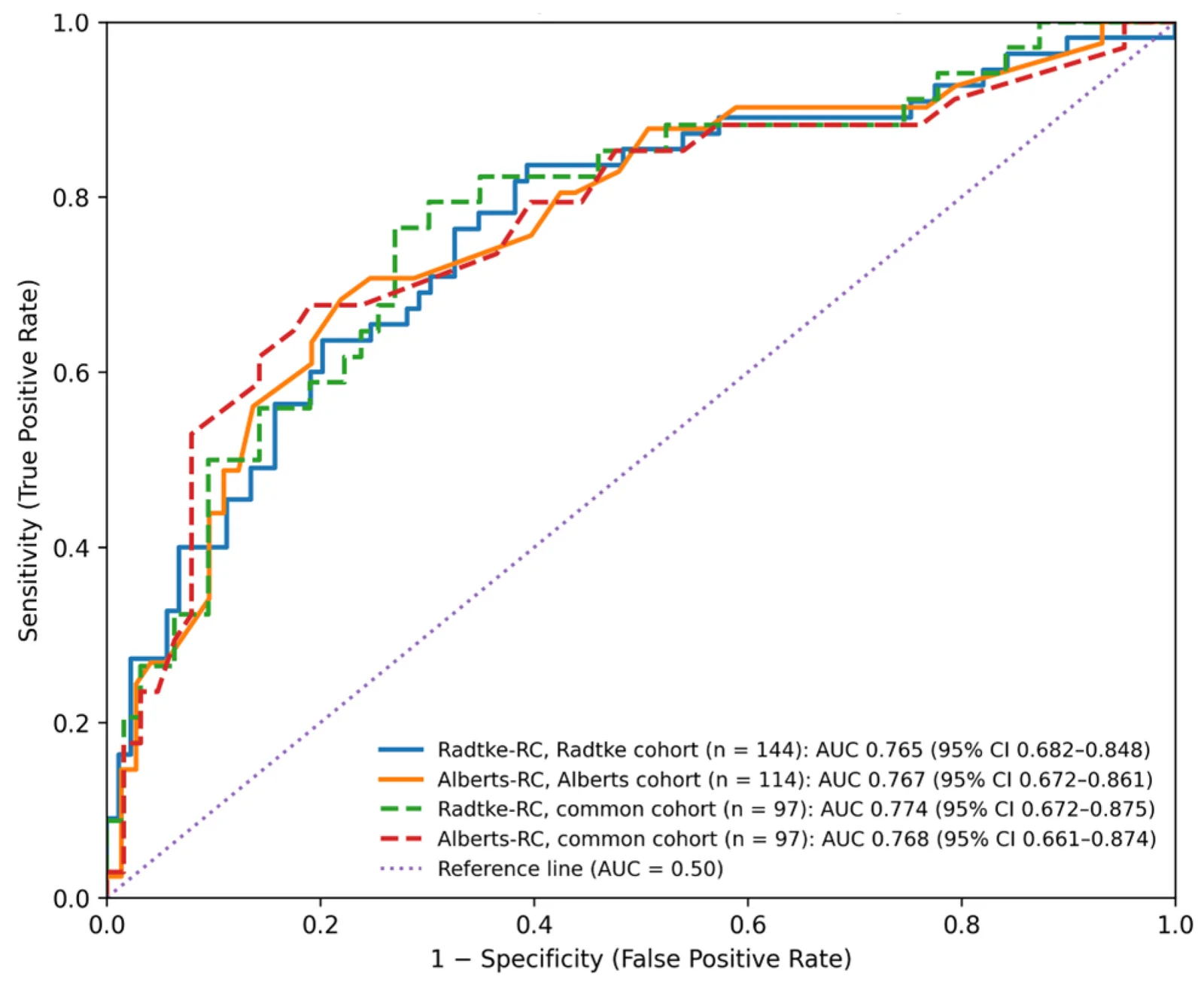
Receiver operating characteristic curves for csPCa prediction in the model-specific and common cohorts. In the model-specific cohorts, the AUC was 0.765 (95% CI 0.682–0.848) for the Radtke-RC (*n* = 144) and 0.767 (95% CI 0.672–0.861) for the Alberts-RC (*n* = 114). In the common biopsy-naïve cohort (*n* = 97), AUCs were 0.774 (95% CI 0.672–0.875) for the Radtke-RC and 0.768 (95% CI 0.661–0.874) for the Alberts-RC. The paired AUC difference in the common cohort was 0.006 (95% CI −0.028 to 0.040; DeLong *p* = 0.726). The model-specific AUCs were estimated in different, partially overlapping populations and were therefore not formally compared with each other. The diagonal line represents no discrimination (AUC = 0.50). Abbreviations: AUC, area under the curve; CI, confidence interval; csPCa, clinically significant prostate cancer; RC, risk calculator.

In the model-specific cohorts, the Radtke-RC achieved an AUC of 0.765 (95% CI 0.682–0.848) and the Alberts-RC an AUC of 0.767 (95% CI 0.672–0.861). In the common biopsy-naïve cohort, AUCs were 0.774 (95% CI 0.672–0.875) for the Radtke-RC and 0.768 (95% CI 0.661–0.874) for the Alberts-RC. The corresponding ROC curves are presented in Figure 2. The paired AUC difference was 0.006 (95% CI −0.028 to 0.040), and DeLong’s test did not detect a statistically significant difference (*p* = 0.726). These findings do not establish equivalence between the calculators.

**Figure 3:**
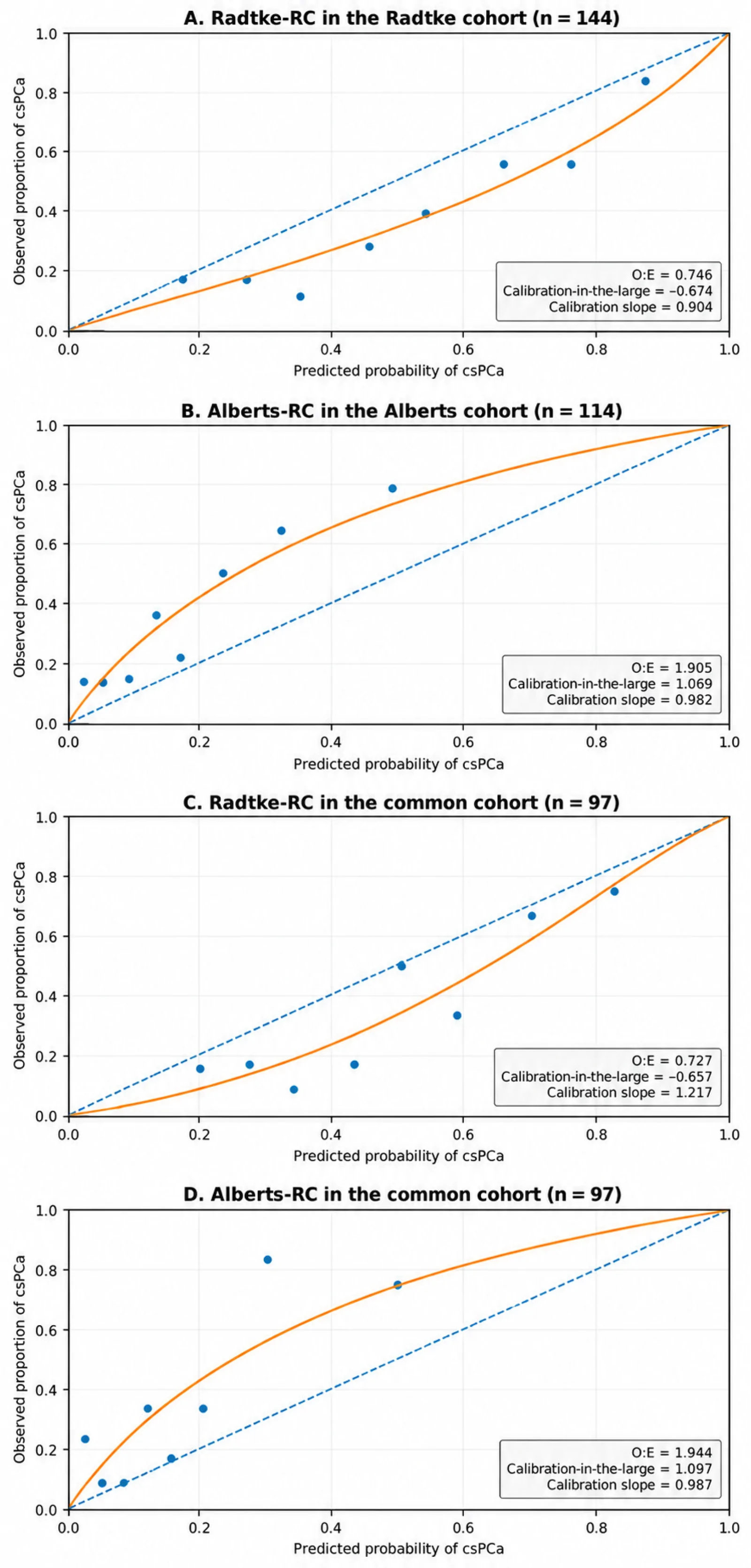
Calibration plots for the Radtke-RC and Alberts-RC in the model-specific and common cohorts. Panels (**A**,**B**) show calibration of the Radtke-RC (*n* = 144) and Alberts-RC (*n* = 114) in their model-specific cohorts; panels (**C**,**D**) show calibration of both calculators in the common biopsy-naïve cohort (*n* = 97). The dashed diagonal line represents perfect calibration, the solid line represents the fitted logistic calibration curve, and the points represent observed event rates by predicted-risk group. Abbreviations: csPCa, clinically significant prostate cancer; RC, risk calculator.

Calibration differed substantially between the models. In the Radtke-RC cohort, observed csPCa prevalence was 38.2% and mean predicted risk was 51.2%, corresponding to an observed-to-expected (O:E) ratio of 0.746. Calibration-in-the-large was −0.674 (95% CI −1.054 to −0.295), and the calibration slope was 0.904 (95% CI 0.527–1.281). In the Alberts-RC cohort, observed prevalence was 36.0% and mean predicted risk was 18.9% (O:E 1.905); calibration-in-the-large was 1.069 and the calibration slope was 0.982. In the common cohort, observed prevalence was 35.1%. Mean predicted risk was 48.2% for the Radtke-RC (O:E 0.727; calibration-in-the-large −0.657, 95% CI −1.114 to −0.200; slope 1.217, 95% CI 0.638–1.797) and 18.0% for the Alberts-RC (O:E 1.944; calibration-in-the-large 1.097, 95% CI 0.631–1.563; slope 0.987, 95% CI 0.481–1.492). Thus, the Radtke-RC showed overall overprediction, whereas the Alberts-RC showed overall underprediction. The corresponding calibration plots are presented in Figure 3.

Predicted-risk distributions differed accordingly. In the common cohort, median predicted risk was 48.3% (IQR 29.9–65.4%) for the Radtke-RC and 14.0% (IQR 6.0–24.0%) for the Alberts-RC; corresponding ranges were 13.0–94.1% and 1.0–84.0%. At thresholds of 12%, 20%, and 30%, the Radtke-RC classified 0/97 (0.0%), 7/97 (7.2%), and 25/97 (25.8%) patients below threshold, respectively. Corresponding values for the Alberts-RC were 42/97 (43.3%), 64/97 (66.0%), and 78/97 (80.4%).

Decision-curve analysis demonstrated strongly threshold-dependent clinical consequences. At 12%, net benefit was 0.2619 for the Radtke-RC and 0.2390 for the Alberts-RC compared with 0.2619 for biopsy-all. Incremental net benefit relative to biopsy-all was 0.0000 (bootstrap 95% CI 0.0000–0.0000) for the Radtke-RC and −0.0230 (95% CI −0.0829 to 0.0286) for the Alberts-RC. The Radtke-RC therefore provided no biopsy reduction at this threshold, whereas the Alberts-RC classified 42 patients below threshold and missed seven csPCa cases, including two ISUP Grade Group ≥ 3 cancers.

At 20%, net benefit was 0.2062 for the Radtke-RC and 0.1985 for the Alberts-RC compared with 0.1881 for biopsy-all. Incremental net benefit was 0.0180 (95% CI 0.0077–0.0309) and 0.0103 (95% CI −0.0722 to 0.0851), respectively. The Radtke-RC classified seven patients below threshold without missing csPCa, whereas the Alberts-RC classified 64 patients below threshold and missed 12 csPCa cases, including two ISUP Grade Group ≥ 3 cancers.

At 30%, net benefit was 0.1237 for the Radtke-RC and 0.1222 for the Alberts-RC compared with 0.0722 for biopsy-all. Incremental net benefit was 0.0515 (95% CI −0.0074 to 0.1046) and 0.0501 (95% CI −0.0648 to 0.1561), respectively.

**Figure 4:**
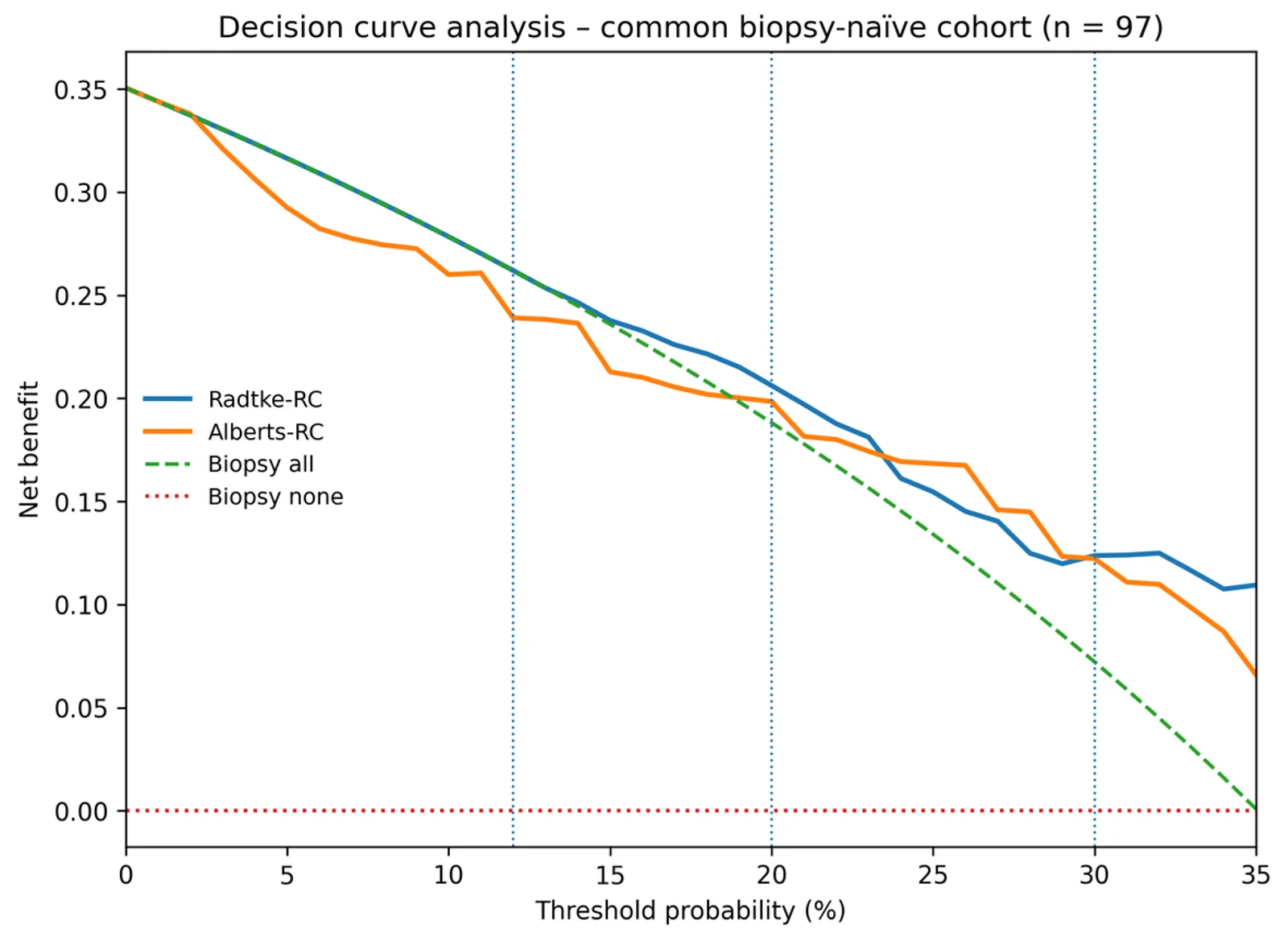
Decision curve analysis of net benefit in the biopsy-naïve common cohort (*n* = 97). The Radtke-RC and Alberts-RC are compared with the default strategies of biopsying all patients and performing no biopsy across threshold probabilities from 0% to 35%. Vertical reference lines indicate the selected threshold probabilities of 12%, 20%, and 30%. The analysis was restricted to patients evaluable for both calculators to permit direct comparison of clinical utility within the same patient population. Bootstrap 95% confidence intervals for incremental net benefit relative to biopsy-all at the selected thresholds are reported in the Results. Abbreviation: RC, risk calculator.

The Radtke-RC classified 25 patients below threshold and missed four csPCa cases, including one ISUP Grade Group ≥ 3 cancer. The Alberts-RC classified 78 patients below threshold and missed 20 csPCa cases, including five ISUP Grade Group ≥ 3 cancers. Of the selected comparisons with biopsy-all, only the Radtke-RC at the 20% threshold had a bootstrap confidence interval for incremental net benefit excluding zero. The corresponding decision curves are presented in Figure 4, while threshold-specific diagnostic performance and clinical consequences are summarized in Table 2.

**Figure 5:**
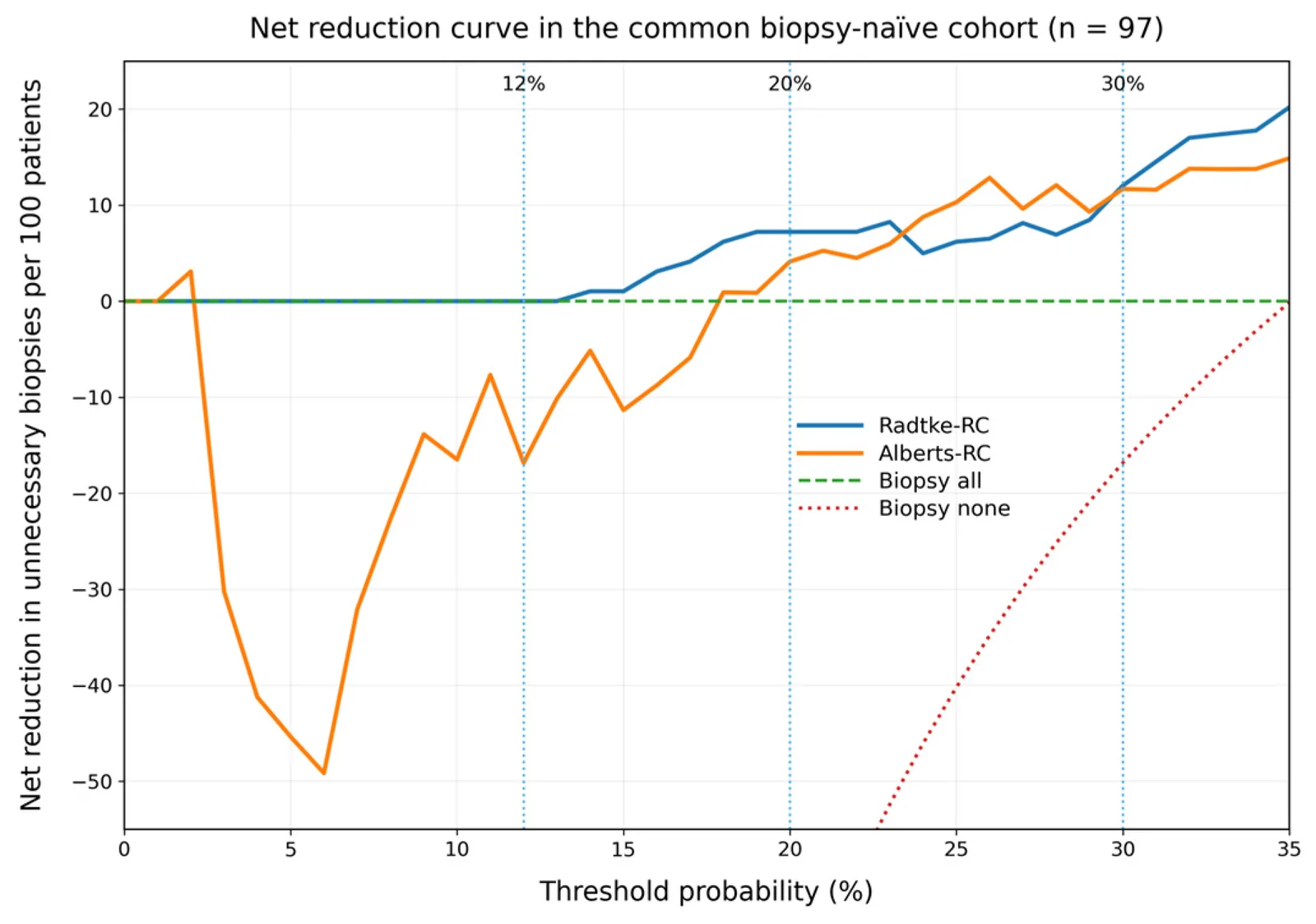
Decision curve analysis of net reduction in unnecessary-biopsy equivalents in the biopsy-naïve common cohort (*n* = 97). The Radtke-RC and Alberts-RC are compared across threshold probabilities from 0% to 35%. Net reduction is expressed as the number of unnecessary-biopsy equivalents per 100 patients relative to the biopsy-all strategy, which has a value of zero by definition. Positive values indicate a decision-analytic reduction relative to biopsy-all, whereas negative values indicate less favourable decision-analytic performance. These quantities are derived from differences in net benefit and do not represent observed numbers of biopsies actually avoided. Vertical reference lines indicate the selected threshold probabilities of 12%, 20%, and 30%. Abbreviation: RC, risk calculator.

Net reductions relative to biopsy-all were 0.0, 7.2, and 12.0 unnecessary-biopsy equivalents per 100 patients for the Radtke-RC at 12%, 20%, and 30%, respectively, compared with −16.8, 4.1, and 11.7 per 100 patients for the Alberts-RC. The corresponding net reduction curves are presented in Figure 5. These values are decision-analytic quantities derived from differences in net benefit and should not be interpreted as observed numbers of biopsies actually avoided; observed numbers of patients classified below threshold and missed cancers are reported separately above and in Table 2.

Sensitivity analyses demonstrated an influence of cohort selection. In the full biopsy-naïve Radtke-RC cohort (*n* = 144), incremental net benefit relative to biopsy-all was −0.0051, 0.0035, and 0.0476 at thresholds of 12%, 20%, and 30%, respectively, corresponding to net reductions of −3.7, 1.4, and 11.1 per 100 patients. Three, 12, and 36 patients were classified below threshold, with one, two, and six csPCa cases missed, respectively. In the mutually exclusive Radtke-only subgroup (*n* = 47), incremental net benefit was −0.0155, −0.0266, and 0.0395, corresponding to net reductions of −11.3, −10.6, and 9.2 per 100 patients. Three, five, and eleven patients were classified below threshold, with one, two, and two csPCa cases missed, respectively. Together with the baseline differences between the common and Radtke-only groups, these findings indicate that restriction to patients evaluable for both calculators influenced the apparent decision-analytic performance at lower thresholds.

Finally, restriction of the common cohort to patients with PI-RADS 4–5 findings (*n* = 84, including 30 patients with csPCa) yielded an AUC of 0.820 for the Radtke-RC and 0.828 for the Alberts-RC. The paired AUC difference was −0.008 (95% CI −0.044 to 0.028), and DeLong’s test again detected no statistically significant difference (*p* = 0.650).

## 4. Discussion

The present study externally validated the Radtke-RC in biopsy-naïve men and the Alberts-RC in eligible patients, with head-to-head comparison restricted to a common biopsy-naïve cohort. No statistically significant difference in discrimination was detected, although the study was not powered to establish equivalence. The models nevertheless showed markedly different calibration and threshold-specific clinical consequences, emphasizing that discrimination, calibration, and decision-analytic performance should be considered jointly [15,20,21].

In the common cohort, the Radtke-RC achieved an AUC of 0.774 and the Alberts-RC an AUC of 0.768. The paired difference was 0.006 (95% CI −0.028 to 0.040), and DeLong’s test did not detect a statistically significant difference. This should not be interpreted as evidence of equivalence, particularly given the 97 patients and 34 csPCa events. The AUC point estimates were close; however, the confidence interval around the paired difference allows for modest differences in either direction. This accords with previous external evaluations [12,13,14,15,16,17,18,24,30].

Calibration provided clinically relevant information beyond discrimination. Observed csPCa prevalence was 35.1%, whereas mean predicted risk was 48.2% for the Radtke-RC and 18.0% for the Alberts-RC. Corresponding O:E ratios of 0.727 and 1.944 indicated overprediction by the Radtke-RC and underprediction by the Alberts-RC. The Alberts-RC calibration slope remained close to unity, suggesting that its miscalibration mainly reflected a shift in absolute risk rather than loss of relative risk ordering. Thus, similar AUCs can translate into different clinical behaviour when fixed thresholds are applied.

Decision curve analysis demonstrated the importance of threshold selection. At 12%, the Radtke-RC classified no patient below threshold, whereas the Alberts-RC classified 42 of 97 patients below threshold but missed seven csPCa cases, including two ISUP Grade Group ≥ 3 cancers. At 20%, the corresponding numbers were seven versus 64, with no csPCa missed by the Radtke-RC and 12 missed by the Alberts-RC. At 30%, 25 and 78 patients, respectively, were classified below threshold, with four and 20 csPCa cases missed. Greater biopsy avoidance should therefore not automatically be interpreted as greater clinical benefit. Bootstrap confidence intervals showed uncertainty in incremental net benefit, particularly for the Alberts-RC. These thresholds should be regarded as illustrative rather than universal cut-offs.

Patient selection materially influenced these findings. Radtke-RC validation was restricted to 144 biopsy-naïve men, whereas the Alberts-RC was applicable in 114 patients, including 97 biopsy-naïve men and 17 with a previous negative biopsy. Only the 97 biopsy-naïve patients were included in the direct comparison. Fifty patients could not be evaluated with the online Alberts calculator because age, prostate volume, or PSA exceeded its permitted input ranges. Among biopsy-naïve men, the 47 patients outside the common cohort were descriptively older and had higher PSA values and larger prostate volumes. Radtke-RC performance at lower thresholds was less favourable in this subgroup, indicating a selection effect.

MRI-related heterogeneity may also have affected model transportability. The csPCa gradient between PI-RADS 3 and 4 was relatively compressed, potentially reducing the contribution of PI-RADS to risk estimates. MRI examinations were interpreted according to PI-RADS version 2.1 by radiologists from University Hospital Salzburg and external outpatient practices without centralized re-review. The exact PI-RADS implementation used in each original development cohort could not be reconstructed with sufficient certainty; nevertheless, differences in PI-RADS assessment and predictor measurement between development and validation settings may have contributed to miscalibration. Restriction to PI-RADS 4–5 patients yielded AUCs of 0.820 and 0.828 for the Radtke-RC and Alberts-RC, respectively, without a statistically significant paired difference.

Pallauf et al. previously evaluated both calculators in men with a previous negative biopsy in a cohort that included University Hospital Salzburg [24]. That cohort was recruited between 2015 and 2017 and did not overlap at patient level with the present 2023–2025 cohort; potential overlap of individual reporting radiologists could not be reliably reconstructed. The present study differs through its biopsy-naïve head-to-head comparison and use of the prevalence-adjusted Radtke implementation. Recent studies and methodological correspondence further emphasize heterogeneity in populations, imaging pathways, calibration, and clinical utility [15,16,17,18,19,21,22,23].

Several limitations should be acknowledged. The retrospective single-centre design and small common cohort limit precision and generalizability, while Alberts-RC input-range restrictions introduced selection. Reader number and experience, the exact method of MRI-derived prostate-volume estimation, and patient-level biopsy route could not be fully reconstructed. Although SWOP predictions required manual web-interface entry, predictor entry and transcription were independently verified by two investigators. The SWOP calculator reports whole-number percentages, limiting granularity around fixed thresholds. Combined targeted and systematic biopsy served as the reference standard; seven of 51 attributable csPCa cases were detected exclusively by systematic biopsy, meaning that complete biopsy avoidance would also have missed these cancers. The study was not prospectively registered. Formal recalibration was not performed because the objective was external validation and updating within the same dataset could produce optimistic results. Larger prospective multicentre studies should evaluate local calibration, model updating, and clinically acceptable decision thresholds before routine implementation.

## 5. Conclusions

No statistically significant difference in discrimination between the Radtke-RC and Alberts-RC was detected; however, the study was not powered to establish equivalence. Despite closely similar point estimates of discrimination, the calculators showed substantially different calibration and threshold-specific clinical consequences in the common biopsy-naïve MRI-selected cohort. These findings highlight the importance of considering calibration, patient selection, and the trade-off between biopsy avoidance and missed csPCa before fixed risk thresholds are applied in clinical practice.

## Figures and Tables

**Table 1:** Baseline demographic, clinical, and radiological characteristics of the Radtke-RC cohort (*n* = 144), Alberts-RC cohort (*n* = 114), and common biopsy-naïve cohort (*n* = 97). Data are presented as mean ± standard deviation, median (interquartile range), or number (percentage), as appropriate. Because the model-specific and common cohorts overlap by design, characteristics are presented descriptively without formal between-cohort hypothesis testing. Abbreviations: csPCa, clinically significant prostate cancer; DRE, digital rectal examination; PI-RADS, Prostate Imaging Reporting and Data System; PSA, prostate-specific antigen; RC, risk calculator.

Characteristic	Radtke-RC Cohort (*n* = 144)	Alberts-RC Cohort (*n* = 114)	Common Cohort (*n* = 97)
**Clinical characteristics**			
Age, years	68.7 ± 8.5	64.5 ± 5.5	64.3 ± 5.5
PSA, ng/mL	7.40 (5.42–10.17)	6.98 (5.00–9.88)	6.70 (5.00–9.80)
Prostate volume, mL	49.5 (37.0–68.3)	46.0 (35.3–60.0)	46.0 (35.0–60.0)
**Digital rectal examination**			
Non-suspicious DRE, *n* (%)	104 (72.2)	83 (72.8)	71 (73.2)
Suspicious DRE, *n* (%)	40 (27.8)	31 (27.2)	26 (26.8)
**Biopsy status**			
Biopsy-naïve, *n* (%)	144 (100.0)	97 (85.1)	97 (100.0)
Previous negative biopsy, *n* (%)	0 (0.0)	17 (14.9)	0 (0.0)
**MRI findings/PI-RADS distribution**			
PI-RADS 1, *n* (%)	2 (1.4)	1 (0.9)	1 (1.0)
PI-RADS 3, *n* (%)	15 (10.4)	14 (12.3)	12 (12.4)
PI-RADS 4, *n* (%)	79 (54.9)	67 (58.8)	56 (57.7)
PI-RADS 5, *n* (%)	48 (33.3)	32 (28.1)	28 (28.9)
**Outcome**			
csPCa, *n* (%)	55 (38.2)	41 (36.0)	34 (35.1)

**Table 2:** Clinical consequences of applying the Radtke-RC and Alberts-RC at selected threshold probabilities in the biopsy-naïve common cohort (*n* = 97). Patients below threshold represent patients who would be classified to avoid biopsy under the respective model-based strategy. Missed csPCa denotes ISUP Grade Group ≥ 2 disease among patients classified below threshold; ISUP Grade Group ≥ 3 cancers are additionally reported. Sensitivity, specificity, and negative predictive value are calculated for detection of csPCa. NPV was not estimable when no patient was classified below threshold. Abbreviations: csPCa, clinically significant prostate cancer; ISUP, International Society of Urological Pathology; NPV, negative predictive value; RC, risk calculator.

Risk Calculator	Thresh-Old	Below Threshold, *n* (%)	Missed csPCa, *n*	Missed ISUP ≥ 3, *n*	Sensitivity, %	Specificity, %	NPV, %
Radtke-RC	12%	0 (0.0)	0	0	100.0	0.0	—
Radtke-RC	20%	7 (7.2)	0	0	100.0	11.1	100.0
Radtke-RC	30%	25 (25.8)	4	1	88.2	33.3	84.0
Alberts-RC	12%	42 (43.3)	7	2	79.4	55.6	83.3
Alberts-RC	20%	64 (66.0)	12	2	64.7	82.5	81.3
Alberts-RC	30%	78 (80.4)	20	5	41.2	92.1	74.4

## Data Availability

The participant-level data underlying this study are not publicly available because they contain pseudonymized clinical information and are subject to applicable data-protection and institutional requirements. Pseudonymized data and the analysis code may be made available by the corresponding author upon reasonable request, subject to institutional approval and compliance with applicable ethical and data-protection requirements.

## Abbreviations

AUC	Area under the receiver operating characteristic curve
CI	Confidence interval
csPCa	Clinically significant prostate cancer
DCA	Decision curve analysis
DRE	Digital rectal examination
ERSPC	European Randomized Study of Screening for Prostate Cancer
ISUP	International Society of Urological Pathology
mpMRI	Multiparametric magnetic resonance imaging
MRI	Magnetic resonance imaging
PCa	Prostate cancer
PCPT	Prostate Cancer Prevention Trial
PI-RADS	Prostate Imaging Reporting and Data System
PSA	Prostate-specific antigen
RC	Risk calculator
ROC	Receiver operating characteristic
SD	Standard deviation
SWOP	Stichting Wetenschappelijk Onderzoek Prostaatkanker
Alberts-RC	Alberts Prostate Cancer Risk Calculator
Radtke-RC	Radtke Prostate Cancer Risk Calculator

## References

[1] Bray F., Laversanne M., Sung H., Ferlay J., Siegel R.L., Soerjomataram I., Jemal A. (2024). Global cancer statistics 2022: GLOBOCAN estimates of incidence and mortality worldwide for 36 cancers in 185 countries. CA Cancer J. Clin..

[2] Loeb S., Bjurlin M.A., Nicholson J., Tammela T.L., Penson D.F., Carter H.B., Carroll P., Etzioni R. (2014). Overdiagnosis and overtreatment of prostate cancer. Eur. Urol..

[3] Drost F.-J.H., Osses D.F., Nieboer D., Steyerberg E.W., Bangma C.H., Roobol M.J., Schoots I.G. (2019). Prostate MRI, with or without MRI-targeted biopsy, and systematic biopsy for detecting prostate cancer. Cochrane Database Syst. Rev..

[4] Turkbey B., Rosenkrantz A.B., Haider M.A., Padhani A.R., Villeirs G., Macura K.J., Tempany C.M., Choyke P.L., Cornud F., Margolis D.J. (2019). Prostate Imaging Reporting and Data System Version 2.1: 2019 update of Prostate Imaging Reporting and Data System Version 2. Eur. Urol..

[5] Deniffel D., Healy G.M., Dong X., Ghai S., Salinas-Miranda E., Fleshner N., Hamilton R., Kulkarni G., Toi A., van der Kwast T. (2021). Avoiding unnecessary biopsy: MRI-based risk models versus a PI-RADS and PSA density strategy for clinically significant prostate cancer. Radiology.

[6] Thompson I.M., Ankerst D.P., Chi C., Goodman P.J., Tangen C.M., Lucia M.S., Feng Z., Parnes H.L., Coltman C.A. (2006). Assessing prostate cancer risk: Results from the Prostate Cancer Prevention Trial. J. Natl. Cancer Inst..

[7] Roobol M.J., Steyerberg E.W., Kranse R., Wolters T., Bergh R.C.v.D., Bangma C.H., Schröder F.H. (2010). A risk-based strategy improves prostate-specific antigen-driven detection of prostate cancer. Eur. Urol..

[8] Trottier G., Roobol M.J., Lawrentschuk N., Boström P.J., Fernandes K.A., Finelli A., Chadwick K., Evans A., van der Kwast T.H., Toi A. (2011). Comparison of risk calculators from the Prostate Cancer Prevention Trial and the European Randomized Study of Screening for Prostate Cancer in a contemporary Canadian cohort. BJU Int..

[9] Louie K.S., Seigneurin A., Cathcart P., Sasieni P. (2015). Do prostate cancer risk models improve the predictive accuracy of PSA screening? A meta-analysis. Ann. Oncol..

[10] Radtke J.P., Wiesenfarth M., Kesch C., Freitag M.T., Alt C.D., Celik K., Distler F., Roth W., Wieczorek K., Stock C. (2017). Combined clinical parameters and multiparametric magnetic resonance imaging for advanced risk modeling of prostate cancer: Patient-tailored risk stratification can reduce unnecessary biopsies. Eur. Urol..

[11] Alberts A.R., Roobol M.J., Verbeek J.F., Schoots I.G., Chiu P.K., Osses D.F., Tijsterman J.D., Beerlage H.P., Mannaerts C.K., Schimmöller L. (2019). Prediction of high-grade prostate cancer following multiparametric magnetic resonance imaging: Improving the Rotterdam European Randomized Study of Screening for Prostate Cancer risk calculators. Eur. Urol..

[12] Lee H.J., Lee A., Yang X.Y., Law Y.M., Huang H.H., Lau W.K., Lee L.S., Ho H.S., Cheng C.W., Yuen J.S. (2021). External validation and comparison of magnetic resonance imaging-based predictive models for clinically significant prostate cancer. Urol. Oncol..

[13] Saba K., Wettstein M.S., Lieger L., Hötker A.M., Donati O.F., Moch H., Ankerst D.P., Poyet C., Sulser T., Eberli D. (2020). External validation and comparison of prostate cancer risk calculators incorporating multiparametric magnetic resonance imaging for prediction of clinically significant prostate cancer. J. Urol..

[14] Püllen L., Radtke J.P., Wiesenfarth M., Roobol M.J., Verbeek J.F., Wetter A., Guberina N., Pandey A., Hüttenbrink C., Tschirdewahn S. (2020). External validation of novel magnetic resonance imaging-based models for prostate cancer prediction. BJU Int..

[15] Patel H.D., Remmers S., Ellis J.L., Li E.V., Roobol M.J., Fang A.M., Davik P., Rais-Bahrami S., Murphy A.B., Ross A.E. (2024). Comparison of Magnetic Resonance Imaging-Based Risk Calculators to Predict Prostate Cancer Risk. JAMA Netw. Open.

[16] Diamand R., Guenzel K., Jabbour T., Baudewyns A., Bourgeno H.-A., Lefebvre Y., Ferriero M., Simone G., Fourcade A., Fournier G. (2024). External validation and comparison of magnetic resonance imaging-based risk prediction models for prostate biopsy stratification. World J. Urol..

[17] Wei G., Kelly B.D., Timm B., Perera M., Lundon D.J., Jack G., Bolton D.M. (2021). Clash of the calculators: External validation of prostate cancer risk calculators in men undergoing mpMRI and transperineal biopsy. BJUI Compass.

[18] Williams N., Remmers S., Jay A., Santoro K., O’Callaghan M. (2025). External Validation of MRI-Based Prostate Cancer Risk Calculators in an Australian Cohort. ANZ J. Surg..

[19] Abbadi A., Eklund M., Lantz A., Discacciati A., Björnebo L., Palsdottir T., Engel J.C., Jäderling F., Falagario U., Grönberg H. (2025). Evaluating the performance of existing tools to predict clinically significant prostate cancer in men with indeterminate lesions on biparametric MRI and development of a novel multiplex model: A prospective cohort study. eClinicalMedicine.

[20] Vickers A.J., van Calster B., Steyerberg E.W. (2019). A simple, step-by-step guide to interpreting decision curve analysis. Diagn. Progn. Res..

[21] Courtney O’Toole C., Boakye N.F., Hannigan A., Jalali A. (2026). Clinical impact of MRI-based risk calculators for prostate cancer diagnosis: A systematic review and meta-analysis. Prostate Cancer Prostatic Dis..

[22] De Riese W.T.W., De Souza A., Renstrom C. (2026). Methodological and clinical considerations of MRI-based prostate cancer risk calculators. Prostate Cancer Prostatic Dis..

[23] O’TOole C.C., Boakye N.F., Hannigan A., Jalali A. (2026). Response to de Riese et al. regarding “methodological and clinical considerations of MRI-based prostate cancer risk calculators”. Prostate Cancer Prostatic Dis..

[24] Pallauf M., Steinkohl F., Zimmermann G., Horetzky M., Rajwa P., Pradere B., Lindner A.K., Pichler R., Kunit T., Shariat S.F. (2022). External validation of two mpMRI-risk calculators predicting risk of prostate cancer before biopsy. World J. Urol..

[25] van Leenders G.J., van der Kwast T.H., Grignon D.J., Evans A.J., Kristiansen G., Kweldam C.F., Litjens G., McKenney J.K., Melamed J., Mottet N. (2020). The 2019 International Society of Urological Pathology (ISUP) Consensus Conference on Grading of Prostatic Carcinoma. Am. J. Surg. Pathol..

[26] Radtke J.P., Giganti F., Wiesenfarth M., Stabile A., Marenco J., Orczyk C., Kasivisvanathan V., Nyarangi-Dix J.N., Schütz V., Dieffenbacher S. (2019). Prediction of significant prostate cancer in biopsy-naïve men: Validation of a novel risk model combining MRI and clinical parameters and comparison to an ERSPC risk calculator and PI-RADS. PLoS ONE.

[27] Rotterdam Prostate Cancer Risk Calculator. Seven Prostate Cancer Risk Calculators. https://www.prostatecancer-riskcalculator.com/seven-prostate-cancer-risk-calculators.

[28] DeLong E.R., DeLong D.M., Clarke-Pearson D.L. (1988). Comparing the areas under two or more correlated receiver operating characteristic curves: A nonparametric approach. Biometrics.

[29] Collins G.S., Reitsma J.B., Altman D.G., Moons K.G.M., members of the TRIPOD group (2015). Transparent Reporting of a Multivariable Prediction Model for Individual Prognosis or Diagnosis (TRIPOD): The TRIPOD Statement. Eur. Urol..

[30] Doan P., Graham P., Lahoud J., Remmers S., Roobol M.J., Kim L., Patel M.I. (2021). A comparison of prostate cancer prediction models in men undergoing both magnetic resonance imaging and transperineal biopsy: Are the models still relevant?. BJU Int..

